# Pharmacological Modulation of BET Family in Sepsis

**DOI:** 10.3389/fphar.2021.642294

**Published:** 2021-03-11

**Authors:** Nian Wang, Runliu Wu, Paul B. Comish, Rui Kang, Daolin Tang

**Affiliations:** Department of Surgery, UT Southwestern Medical Center, Dallas, TX, United States

**Keywords:** bromodomain and extra-terminal, inhibitor, innate immune, inflammation, sepsis

## Abstract

The Third International Consensus Definitions for Sepsis and Septic Shock (Sepsis 3.0) recommended defining sepsis as a life-threatening organ dysfunction caused by the host's uncontrolled response to infection. The bromodomain and extra-terminal (BET) protein family (such as BRD2, BRD3, and BRD4), an epigenetic regulator of gene transcription, has recently been recognized as a significant septic regulator of inflammation and immune response, including cytokine and chemokine production. Mechanistically, the two N-terminal conserved tandem bromodomains (namely the first bromodomain [BD1] and the second bromodomain [BD2]) favor the binding of BETs to acetylated histones or transcription factors, thereby initiating gene transcription machinery after CycT1 and CDK9 (also known as P-TEFb) are recruited to gene promoters to phosphorylate RNA pol II. Notably, BD1 and BD2 are not functionally redundant because they have different target genes in innate immune cells. Small-molecule BET inhibitors (BETis) for different BDs, such as I-BET, JQ1, I-BET151, apabetalone, RVX-297, and dBET1 have shown promising therapeutic effects in experimental sepsis models. This mini-review summarizes the emerging roles of BETs and the applications of BETis in sepsis, discusses the existing shortcomings of BETis, and introduces possible future research directions in this area.

## Introduction

Sepsis is a medical condition driven by an unrestricted host response to infection and subsequent multiple organ dysfunction or failure (Singer et al., 2016). While bacterial infections are considered to be the most common causes of sepsis, other pathogen infections, such as fungus, virus, and parasite, also initiate sepsis (Dolin et al., 2019). Despite considerable medical advances in recent years, especially intensive care support and the application of antibiotics, the mortality rate of patients with sepsis remains high (>25%). Once patients develop septic shock with multiple organ dysfunction syndrome (MODS), the mortality rate can reach as high as 70% (Rudd et al., 2020). Thus, sepsis is still a big challenge in modern medicine.

The pathophysiology of sepsis is complex and involves multiple steps (Kang et al., 2018; Chen et al., 2019). Cytokine storm, a well-established mechanism for sepsis, results in uncontrolled inflammatory responses (Chousterman et al., 2017). However, antibody drugs targeting cytokines (e.g., tumor necrosis factor [TNF] and interleukin 6 [IL6]), inflammatory pathways (e.g., toll like receptor 4 [TLR4]), or endotoxin, as well as empiric antibiotic therapies have little or disappointing benefit for patients with sepsis (Chaudhry et al., 2013). Since the production of inflammation and immune response genes involved in sepsis is strictly controlled at the transcriptional, posttranscriptional, translational, and posttranslational levels, targeting these regulatory pathways may reasonably provide potential treatment strategies for sepsis (Carson et al., 2011; Vachharajani and McCall, 2019).

The epigenome describes all heritable chemical modifications added to DNA and histone proteins, which regulates the transcription of genes in the genome without affecting the DNA sequence (Zhang and Cao, 2019). The main epigenetic machinery includes DNA alterations (e.g., methylation and oxidation), histone modifications (e.g., acetylation, ubiquitination, phosphorylation, and methylation), and microRNA regulations (Topper et al., 2020). As a reversible chromatin modification, histone acetylation is affected by histone acetyltransferases (HATs, “epigenetic writer”) and histone deacetylases (HDACs, “epigenetic eraser”). After acetylation, the acetylated lysine within the N-terminal tail protruding from the histone core of the nucleosome is recognized by the epigenetic reader and bound to their specific structural domains (e.g., bromodomain), leading to the activation of nuclear transcription factor and subsequent gene transcription (Peserico and Simone, 2011). Hence, epigenetic readers are molecular gatekeepers of gene expression and become promising drug targets of diseases.

Bromodomain is an evolutionarily conserved protein-protein interaction module, which comprises approximately 110 amino acids that recognize acetylated lysine residues within histones and other proteins. 61 bromodomain modules, including bromodomain and extra-terminal domain (BET) family (Filippakopoulos et al., 2012), have been identified in various species. The BET protein family, including BRD2, BRD3, BRD4, and BRDT, plays a complex role in coordinating innate immune responses through epigenetic regulation of gene transcription.

**FIGURE 1:**
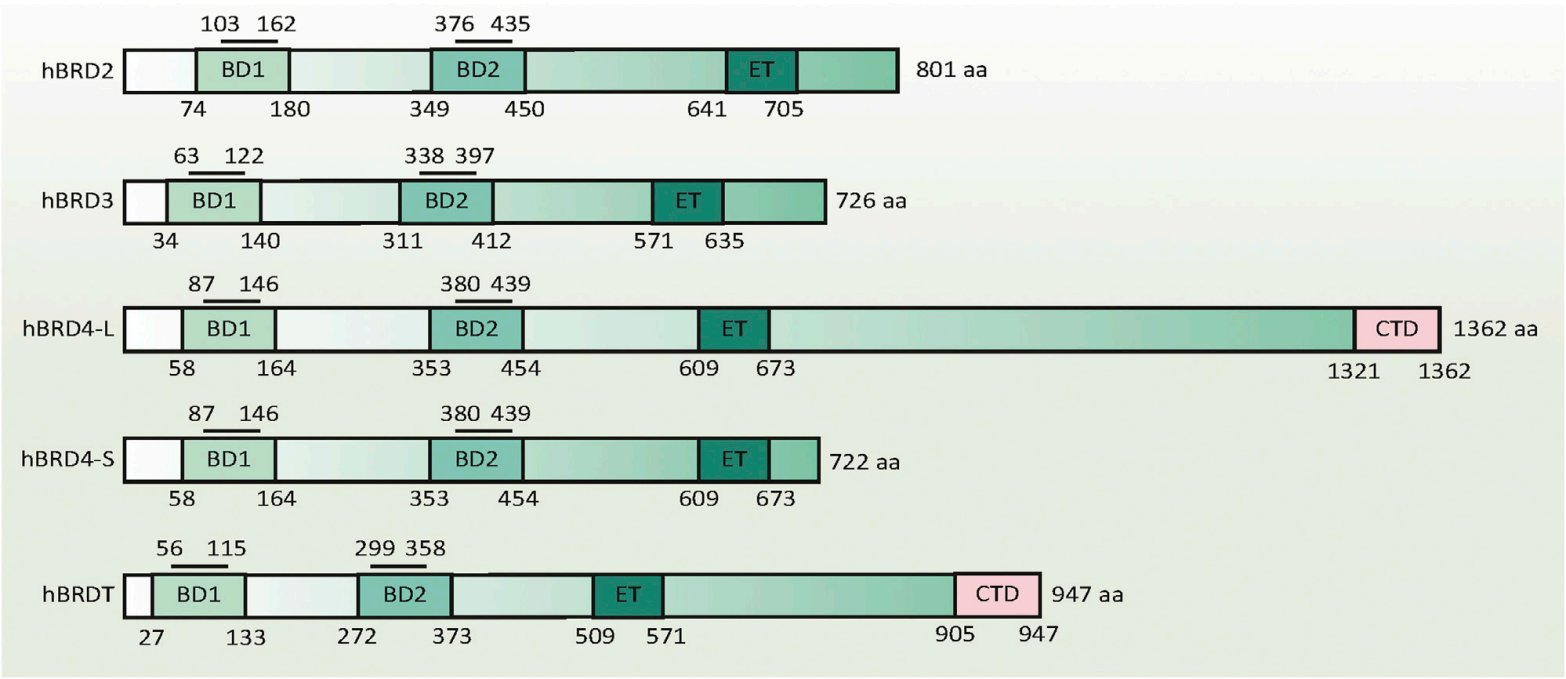
Domain structure of human BET family proteins. The numbers below the columns indicate the amino acid boundaries of each domain in each BET. The numbers above the column indicate the amino acid boundaries of the acetyl-lysine binding sites in the BD1 and BD2 domains. The amino acid sequence alignment is based on published information, using the following accession numbers retrieved from the GenBank database: hBRD2, NM_005104; hBRD3, NM_007371.4; hBRD4-L, NM_058243; hBRD4-S, NM_014299; and hBRDT, NM_001242805. Abbreviations BD1, the first bromodomain; BD2, the second bromodomain; BRD, bromodomain protein (numbers 2-4); CTD, C-terminal domain; ET, extra-terminal domain

**FIGURE 2:**
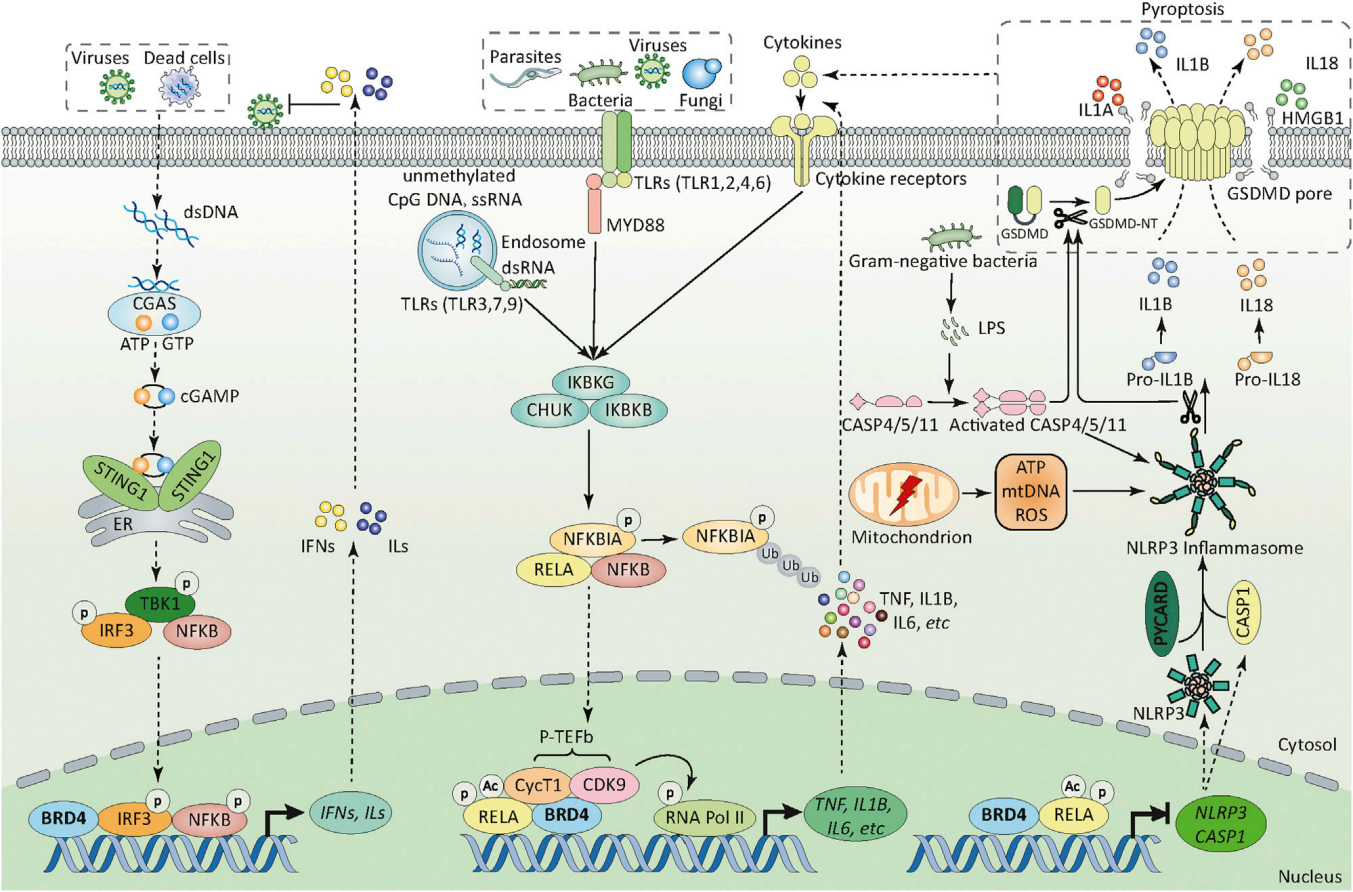
BRD4 in innate immunity. BRD4 can sense and orchestrate different PRR-mediated innate immunity through transcriptional regulation. First, upon pathogen infection (e.g., bacteria, fungi, parasites, and viruses), BRD4 regulates the activation of different TLR-mediated NF-κB pathways through promoting the phosphorylation of RELA at Ser276 and its acetylation at Lys310. In addition, BRD4 recruits CycT1 and CDK9 (also known as P-TEFb) to the promoters of target genes which can phosphorylate RNA pol II at serine 2 and 5, thereby initiating gene transcriptional machinery. Second, BRD4 can promote or inhibit the activation of NLRP3 inflammasome pathway through transcriptional regulation of NRLP3 and CASP1 in a RELA-dependent manner. BRD4-mediated NLRP3 expression in innate immunity is context dependent. Third, cytoplasmic DNA derived from viruses and dead cells activates CGAS and produces endogenous cyclic dinucleotide cGAMP, which further binds to STING1 located in the endoplasmic reticulum, then induces the dimerization and translocation of STING1 from the ER to the perinuclear region. During trafficking, STING1 recruits and activates TBK1, stimulates the phosphorylation and nuclear translocation of IRF3, and to a lesser extent NFKB1, which leads to the production of type 1 IFN and other inflammatory cytokines (e.g., TNF and IL6).; Abbreviations: ATP, adenosine triphosphate; BRD4, bromodomain containing 4; CASP1, caspase 1; CASP4, caspase 4; CASP5, caspase 5; CASP11, caspase 11; CDK9, cyclin-dependent kinase 9; cGAMP, cyclic GMP-AMP; CGAS, cyclic GMP-AMP synthase; CHUK, component of inhibitor of nuclear factor kappa B kinase complex; CycT1, cyclin T1; dsDNA, double-stranded DNA; dsRNA, double-stranded RNA; ER, endoplasmic reticulum; HMGB1, high mobility group box 1, IFN, interferon; IKBKB, inhibitor of nuclear factor kappa B kinase regulatory subunit beta; IKBKG, inhibitor of nuclear factor kappa B kinase regulatory subunit gamma; ILs, interleukins; IL1A, interleukin 1 alpha; IL1B, interleukin 1 beta; IL18, interleukin 18; IL6, interleukin 6;IRF3, interferon regulatory factor 3; GSDMD, gasdermin D; GSDMD-NT, gasdermin D N-terminal domain; GTP, guanosine triphosphate; LPS, lipopolysaccharide; mtDNA, mitochondrial DNA; MYD88, myeloid differentiation primary response 88 (MYD88); NFKBIA, NFKB inhibitor alpha; NFKB1, nuclear factor kappa B subunit 1; NLRP3, NLR family pyrin domain containing 3; p-TEFb, positive transcription elongation factor b; PYCARD, PYD and CARD domain containing; RELA, RELA proto-oncogene; RNA pol II, RNA polymerase II; ROS, reactive oxygen species; ssRNA, single-stranded RNA; STING1, stimulator of interferon response cGAMP interactor 1; TBK1, TANK binding kinase 1; TLR, toll-like receptor (numbers 1-9); TNF, tumor necrosis factor.

In this mini-review, we not only outline the structure and function of BET family in innate immunity (Figure 1 and Figure 2), but also summarize the application of various BET inhibitors (BETis) in experimental models of sepsis (Table 1). These emerging knowledge may help to further develop novel anti-sepsis strategies.

**TABLE 1:** BET inhibitors in sepsis.

Inhibitor	Target	IC50	Septic animal or cell model	Dose of BETi	Administration method	Target molecule and main action	Chemical structure	Ref
I-BET (GSK525762A)	BRD4 (BD1)	32.5–42.5 nM	Cecal ligation puncture (CLP)-treated mice	30 mg/kg	Intravenous injection (i.v.), twice daily for 2 days	Downregulation of IL6, IFNB1, IL1B, IL12A, CXCL9 and CCL12, *etc.*		Nicodeme et al. (2010)
			Heat-killed Salmonella typhimurium (strain IR71, 5×10^9^/kg, i.v.)-treated mice	30 mg/kg	i.v., twice daily for 2 days		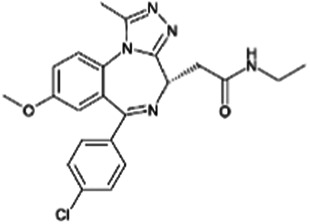	
			LPS (5 mg/kg, intraperitoneal injection[i.p.])-treated mice	30 mg/kg	i.v., twice daily for 2 days			
			LPS (100 ng/ml)-induced mouse immortalized bone marrow-derived macrophages (iBMDMs)	5 μM	Pretreatment (at 30 min before LPS stimulation)			
(+)-JQ1	BRD2, BRD4 (BD1 and BD2)	77/33 nM (BD1 and BD2)	LPS (20 mg/kg, i.p.)-treated mice	50 mg/kg	i.p., at 2 h before and 24 h after LPS injection	Downregulation of IL6 and TNF		Belkina et al. (2013)
			*L. monocytogenes* (strain LO28, multiplicity of infection [MOI] = 20)-infected mouse iBMDMs (4 h)	250 nM	Pretreatment (at 1 h before infection and left in the culture medium during infection	Downregulation of NOS2, IL6, IL1RN, TNF, SLAMF1, IRF8, MXD1, IL19, IFITM1, GBP2, IFNB, DUSP2, *etc.*	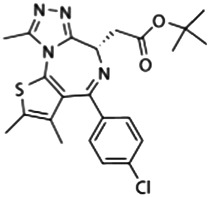	Wienerroither et al. (2014)
			*L. monocytogenes* (strain LO28, MOI = 20)-infected mouse (24 h)	50 mg/kg	i.p.			
			LPS (100 ng/ml)-treated mouse primary astrocytes (24 h)	100 nM	Pretreatment (at 30 min before LPS stimulation)	Downregulation of SERPINE1		Choi et al. (2015))
I-BET151 (GSK1210151A)	BRD2, BRD3, and BRD4 (BD1 and BD2)	0.5 μM (BRD2), 0.25 μM (BRD3), and 0.79 μM (BRD4)	LPS (20 mg/kg, i.p.)-treated mice	10 mg/kg	i.v., at 60 min before or 90 min after LPS challenge	Downregulation of IL6	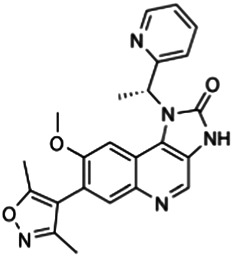	Seal et al. (2012)
Apabetalone (RVX-208 or RVX-000222)	BRD4 (BD2)	87 ± 10 μM (BD1); 0.51 ± 0.041 mM (BD2)	LPS (10 μg, i.p.)-induced endotoxemic mice	150 mg/kg	Gavage for 7 days	Downregulation of APCS, A2M, CD14, and CCR2	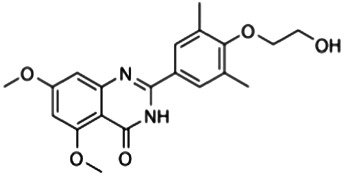	Wasiak et al. (2020)
RVX-297	BRD2 and BRD4 (BD2)	30 and 80 nM,	LPS (5 or 10 μg, i.p.)-induced endotoxemic mice	75 mg/kg	Gavage at 4 h before LPS stimulation and again when stimulated with LPS	Downregulation of IL6, IL17, CSF2, CCL2, IL2, TNF, and IFNG	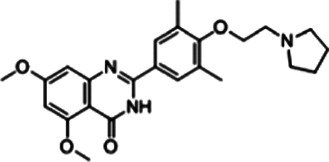	Jahagirdar et al. (2017)
			LPS (1 µg/ml)-induced mouse BMDMs (3 h)	10 μM	Combined treatment with LPS			
dBET1	BRD2 and BRD4	20 nM	LPS (10 ng/ml)-treated microglia (24 h)	1 μM	Pretreatment (at 1h before LPS stimulation)	Downregulation of NOS2, IL1B, TNF, CCL2, IL6, PTGS2, and MMP9	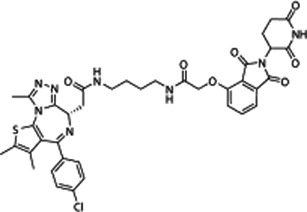	DeMars et al. (2018)

## Characteristics of BETs in Innate Immunity

### Expression of BETs

Normally, the expression of BRDT is restricted to the testis, while the expression of BRD2, BRD3, or BRD4 is commonly found in the nucleus of other cells (including immune cells). Under pathological conditions, the expression of BETs may be further changed (upregulation or downregulation) to meet the requirements for orchestrating a genetic regulatory response (Bachtel et al., 2019; Hong et al., 2020). In the case of sepsis, according to the type of pathogen infection, the expression of BRD2, BRD3 or BRD4 shows heterozygosity and diversity in immune cells. For example, BRD4 expression can be up-regulated, down-regulated or unchanged in activated macrophages or memory CD4^+^ T cells during virus infection (Bachtel et al., 2019). The expression profile of the BETs may not be a good biomarker of sepsis, although they have functions in innate immunity (discussed later). While several miRNAs (e.g., miR-218-5p, miR-29a, and miRNA-302e) act as the posttranscriptional regulators of BRD4 expression (Lin et al., 2018; Li et al., 2019; Tang et al., 2019), the transcriptional regulatory mechanisms controlling the expression of BETs remain obscure.

### Structure of BETs

Although the amino acid length of each BET is different, they have a similar protein secondary structure: two N-terminal conserved tandem bromodomains (namely the first bromodomain [BD1] and the second bromodomain [BD2]) and a unique C-terminal extraterminal (ET) domain. These BDs contain four alpha helices, which are separated by a variable loop region to form a central hydrophobic cavity. Acetylated lysine residues in histone tails and other proteins (e.g., RELA proto-oncogene [RELA] and tumor protein p53 [TP53]) are recognized through this central hydrophobic pocket by anchoring to a conserved asparagine residue (Filippakopoulos et al., 2012). Unlike other bromodomain proteins, BETs prefer to bind to diacetylated lysine residues with an optimal spacing of two amino acids (Kac-XX-Kac) closely located in the protein sequence. The acetyl-lysine binding sites in BD1 and BD2 containing 59 amino acids have unique binding selectivity and are not redundant in function (Figure 1). BD1 binds chromatin components, especially diacetylated residues on histone H4 (e.g., H4K5Ac, H4K8Ac, and H4K12Ac). BD2 accommodates a wide range of diacetylated residues and facilitates the recruitment of BETs to induce gene transcription, which is relatively more permissive (Moriniere et al., 2009; Gamsjaeger et al., 2011).

As for the C-terminal ET domain in each BET, they contain a helical architecture, including an acidic surface, shaped in a continuous ridge. Because the ET domain is responsible for the interaction between proteins, BETs act as scaffold proteins for the recruitment of transcription factors and coactivators. Other domains, such as motif B and Ser/Glu/Asp-rich region (SEED), are conserved in the C-terminal moiety of each BET, whereas the C-terminal domain (CTD) and motif A are not present in every BET (Florence and Faller, 2001). This may be the structural basis for distinguishing the functional differences of each BET. Intriguingly, since the regions in BETs are weakly reminiscent of kinase motifs, BETs exhibit intrinsic kinase activities, which may initiate gene transcription by directly phosphorylating RNA polymerase II (Pol II) at serine 2 and 5 (Denis et al., 2000; Devaiah et al., 2012). Notably, the kinase motifs of BETs lack homology with other known kinase domains. Given this, it is necessary to develop specific BETis targeting kinase activity in the future.

### Function of BETs

BETs not only function as scaffolds to recruit different transcription factors (e.g., RELA, JUN, and MYC) and transcription elongation complexes (e.g., P-TEFb), but also serves as switches to initiate gene transcription machinery upon the interaction of BDs with acetylated chromatin either at gene promoters or in long range cis regulatory elements (namely “enhancers”) (Filippakopoulos and Knapp, 2014). Consequently, BETs regulate the expression of various immune and inflammatory genes in innate immunity.

One of the key events of infection-related innate immunity is the recognition of evolutionary conserved structures on pathogens (namely pathogen-associated molecular patterns [PAMPs]) through different pattern recognition receptors (PRRs) expressed in immune and non-immune cells. Main PRRs include transmembrane (e.g., toll-like receptors [TLRs]) and intracellular (e.g., nucleotide-binding oligomerization domain (NOD)-like receptors [NLRs]) PRRs (Tang et al., 2012). Infection-mediated tissue damage can further amplify the systemic inflammatory response through the release of endogenous damage-associated molecular patterns (DAMPs) (e.g., high mobility group box 1 [HMGB1] and host DNA) by dead or dying cells (Kang, et al., 2014). In these processes, BET plays a role in coordinating gene transcription mediated by PAMPs or DAMPs, which decides the outcome of infection via interaction with TLR, inflammasome, and DNA sensor pathways (Figure 2).

TLRs are central PRRs responsible for recognizing PAMP to trigger the expression of immune mediators by activating transcription factors, such as nuclear factor kappa B (NFKB) and interferon regulatory factors (IRFs). 10 TLRs (TLR1-10) in humans and 13 TLRs (TLR1-13) in mice have been identified, which show different subcellular localizations and PAMP recognition preferences. TLR-1, -2, -4, -5, and -6 are located at the extracellular surface and bind to the components of microbial cell walls and membranes of pathogens (e.g., lipopolysaccharide [LPS], lipoteichoic acid, and lipoproteins). TLR-3, -7, -8, and -9 are mainly expressed in the endoplasmic reticulum and endosome, and detect microbial nucleic acids, such as double or single-stranded RNA from RNA viruses and DNA presented in bacteria and viruses. BET affects the signal pathway of TLRs through two potential mechanisms. On one hand, BETs directly mediate the transcription upregulation of TLR-2, -4, and -6 genes, thereby activating TLR pathway (Nicodeme et al., 2010; Meng et al., 2014; Zhao et al., 2019). On the other hand, BET may promote or inhibit TLR signaling-mediated gene expression through the modulation of activity of NFKB or IRFs in a context-dependent manner. Although the role of BETs in shaping TLR-1, -2, -3, -4, -6, -7, and -9 signaling has been largely demonstrated, their functions in control of TLR-5, -8, and -10, as well as other transmembrane PRRs, are still a mystery.

The inflammasome machinery is important in the innate immune system, which not only mediates the maturation and release of the interleukin-1 (IL1) family (e.g., IL1B and IL18), but also promotes the activation of caspase-1 (CASP1) or CASP4/5/11 to trigger gasdermin D (GSDMD)-dependent pyroptosis to release DAMPs (e.g., HMGB1). One of the largest subfamilies of inflammasomes is called NLR inflammasomes. According to the structure of N-terminal domain, NLRs are further divided into four subfamilies (namely NLRA, NLRB, NLRC, and NLRP) to recognize PAMPs or DAMPs. Among them, the NLR family pyrin domain containing 3 (NLRP3) inflammasome is best characterized and shows sustained activation in sepsis through canonical CASP1-dependent or non-canonical CASP4/5/11-mediated pathway. Interestingly, BETs confer opposite effects on NRLP3 activation depending on cell types. For example, the inhibition of BRD4 alleviates the inflammatory response by blocking TNF-related NRLP3 activation in rat nucleus pulposus cells (Hong et al., 2020). However, the inhibition of BRD4 prevents proliferation and epithelial mesenchymal transition in kidney cancer cells by increasing RELA-mediated NLRP3 expression and subsequent pyroptosis (Tan et al., 2020). Parallel to membrane TLR4, CASP11 acts as a cytoplasmic receptor for LPS in macrophages to trigger endotoxemia in mice. Defining the role of BETs in the regulation of cytoplasmic LPS signaling may further determine the pathological roles of BETs in bacterial infection.

Another multifunctional regulator of innate immunity is the DNA sensor. In addition to TLR9, a receptor essential for identifying unmethylated CpG DNA, cyclic GMP-AMP synthase (CGAS) plays a broad role in the recognition of various types of DNA and their metabolites produced by microorganisms and hosts. After the endogenous second messenger cyclic GMP-AMP (cGAMP) is synthesized by CGAS, cGAMP binds to stimulator of interferon response cGAMP interactor 1 (STING1, also known as STING or TMEM173), resulting in the transcriptional activation of NFKB1 and IRF3, thereby increasing the production of type I interferon and pro-inflammatory cytokines (Chen et al., 2016). The activation of the CGAS-STING1 pathway is related to BRD4 inhibition-mediated antiviral immunity (Wang et al., 2020), indicating that BRD4 is a repressor of the STING1 pathway. Unlike viral infection, excessive activation of STING1 mediates lethal inflammation and systemic coagulation, leading to bacterial septic shock in mice, partly through the activation of NFKB1 and the inflammasome pathway (Zhang et al., 2020; Zhou et al., 2020). This STING1-dependent inflammatory pathway in sepsis seems to depend on the plasma membrane receptor ALK receptor tyrosine kinase (ALK), not cytoplasmic CGAS (Zeng et al., 2017). On this basis, we urgently need to figure out how BRD4 controls the dual role of STING1 pathway in innate immunity.

### Application of BETis in Sepsis and Septic Shock

According to the structure of BETs, some chemical compounds have been developed to disrupt or compete the binding of BD1 and BD2 to acetylated histones and transcription factors. The first-generation BETis are also considered pan-BETis because they have no selectivity for BD1 and BD2. Since the BDs between different BETs have a high degree of homology, these BETis cannot distinguish individual member of the BETs. While selective BD1-BETis and BD2-BETis show different inhibition activity of BET, BD1-BETis seems to be as effective as pan-BETis in some cases (Gilan et al., 2020). However, a recent study has shown that BD1 is primarily required for steady stage gene expression, whereas both BD1 and BD2 in all BETs are required for acute phase gene expression during inflammation (Gilan et al., 2020). Theoretically, BD2-BETis may be predominantly effective in the treatment of acute inflammation, including the hyperinflammatory state in the early stage of sepsis. Below, we summarize the potential applications of BETis in experimental sepsis (Table 1).

### I-BET

I-BET (also known as I-BET762 or GSK525762A) was discovered in 2010 (Nicodeme et al., 2010). It binds to BD1 of BRD4 at the acetyl-lysine (AcK)-binding pocket, which enables two I-BET molecules to bind to the tandem BDs of BET with high affinity. I-BET can successfully compete with AcK within the recognition pocket of BET. I-BET is highly selective and effectively displaces the tetra-acetylated H4 peptide previously bound to the BET tandem BD. Pre-treatment of bone marrow derived macrophages (BMDMs) with I-BET results in the downregulation of 38 and 151 of the LPS-inducible immune genes (including cytokines and chemokines) at 1 and 4 h, respectively. Moreover, the administration of I-BET protects mice from experimental sepsis caused by endotoxemia, polymicrobial peritonitis, and cecal ligation and puncture (CLP). The myeloid lineage-specific *Brd4* conditional knockout mice (termed *Brd4* CKO) are used to further investigate the pathological effects of BRD4 in sepsis. Surprisingly, *Brd4* CKO mice are resistant to endotoxemia, but are more susceptible to intraperitoneal injection of group B *Streptococcus*-induced infection (Bao et al., 2017), which may result from the compromised innate immune response to clear bacteria *in vivo.* These findings not only demonstrate the importance of BRD4 in innate immunity against bacterial infection, but also highlight an unknown function of BRD4 in regulating the activity of bacterial LPS and non-LPS components.

### JQ1

JQ1 (best known as (+)-JQ1), the most widely used BETi reported in 2010, is a thienotriazolodiazepine which competitively binds to both BD1 and BD2 of BETs with acetylated lysine. It exhibits prominent anti-inflammatory and immunoregulatory activity in endotoxemic mice by reducing the levels of IL6 and TNF, and rescues mice from LPS-induced death (Belkina et al., 2013). In BMDMs infected with heat-killed *L. monocytogenes*, JQ1 also inhibits the expression of cytokines, such as nitric oxide synthase 2 (NOS2), IL6, and interleukin 1 receptor antagonist (IL1RN) (Wienerroither et al., 2014). Moreover, JQ1 inhibits LPS-induced the upregulation of inflammatory cytokines and serpin family E member 1 (SERPINE1) in mouse primary astrocytes through the depletion of BRD2 recruitment and H3K4me3 enrichment at the promoter region of SERPINE1 (Choi et al., 2015). In addition to bacterial infection, JQ1 also inhibits the upregulation of immune genes (e.g., IL1B, IL6, interferon beta 1 [IFNB1], ISG15 ubiquitin like modifier [ISG15], and interferon gamma [IFNG]) during infection with viruses (e.g., *Pseudorabies virus*, *Herpes simplex virus*, and *Ectromelia virus*), fungi (e.g., *Candida albicans* and *Aspergillus fumigatus*), and parasites (e.g., *Schistosoma japonicum*) (Wang et al., 2021; Dominguez-Andres et al., 2019; Wang et al., 2020). Despite its strong anticancer activity, the toxicity and side effects of JQ1 for the treatment of sepsis may not be optimistic, and extensive research is needed.

### I-BET151

I-BET151 (also known as GSK1210151A), which belongs to the quinoline isoxazole BET family bromodomain inhibitors, was developed in 2012 and has good oral bioavailability with a similar effect as I-BET (Seal et al., 2012). The administration of I-BET151 also protects mice from LPS-induced death (endotoxemia). Interestingly, I-BET151 cannot affect LPS-induced TNF production, but significantly inhibits LPS-mediated IL6 production. Since both TNF and IL6 are NFKB target genes, I-BET151 may regulate endotoxemia in an NFKB-independent manner. Another open question is whether I-BET151 can be used to inhibit the signal transducer and activator of transcription 3 (STAT3)-related immune pathway, because the inflammatory mediator IL6 is a well-known activator of the STAT3 pathway.

### Apabetalone and RVX-297

Apabetalone (also known as RVX-208 or RVX-000222), an oral BETi selective for BD2 within BETs, is currently used in phase 3 clinical trials for the treatment of coronary artery disease, diabetes, and chronic kidney failure. In a mouse model of lethal endotoxemia, apabetalone prevents liver damage by inhibiting the expression of alpha-2-macroglobulin and serum amyloid P. In patients with cardiovascular disease, apabetalone provides benefit for limiting chronic cytokine signaling (Wasiak et al., 2020). Thus, this action of apabetalone might also serve as an effective therapy in treating patient with sepsis and MODS, especially sepsis-induced cardiomyopathy. BRD2 is a direct target of apabetalone (Gordon et al., 2020). Since apabetalone can inhibit the expression of angiotensin-converting enzyme 2 (ACE2), the receptor utilized by the SARS-CoV-2 particles to gain entry into human cells, it is becoming a promising drug for the treatment of COVID-19 and concomitant sepsis (Gilham et al., 2020). RVX-297, a 4-quinazolinone derivative related to RVX-208, is two times more selective for BD2 than RVX-208. RVX-297 also decreases the production of multiple cytokines in endotoxemic mice (Jahagirdar et al., 2017), highlighting its potential anti-inflammatory activities in lethal infection.

### dBET1

In recent years, the degradation of BETs using proteolytic targeting chimera (PROTAC) has shown excellent targeting ability and activity. The advantage of BETs degradation rather than inhibition is that it may lead to selective suppression of individual BET-dependent genes. Indeed, PROTAC-based BETis (e.g., dBET1, MZ-1, and ARV-825) exhibit promising immunoregulatory activities in various disease models (Suarez-Alvarez et al., 2017; DeMars et al., 2018; Tsujikawa et al., 2019). DBET1 is a conjugate of (+)-JQ1 and cereblon E3 ubiquitin ligase ligand (phthalimide), which can induce highly selective cereblon-dependent BET degradation *in vitro* and *in vivo*. dBET1 effectively inhibits LPS-induced the expression of proinflammatory factors (e.g., NOS2, prostaglandin-endoperoxide synthase 2 [PTGS2], IL1B, TNF, C-C motif chemokine ligand 2 [CCL2], IL6, and matrix metallopeptidase 9 [MMP9]) in the microglia by degrading BRD2 and BRD4 in a time- and dose-dependent manner (DeMars et al., 2018). These activities make DBET1 a promising drug candidate for the treatment of sepsis. However, since the complete loss of BRD2 and BRD4 is lethal, when DBET1 is administered systemically, uncontrolled degradation of BRDs in normal cells may bring toxicity. Therefore, the therapeutic window and adverse reactions of dBET1 needs to be carefully defined in future studies.

## Conclusion and Outlook

Sepsis and septic shock cause more than 11 million deaths each year (Rudd et al., 2020), which is currently compounded by the COVID-19-related septic deaths (Tang et al., 2020). Because of their transcriptional control of immune and inflammatory genes, BETs have become druggable targets for the treatment of diseases, including sepsis (Wang et al., 2021). Although the existing preclinical data seems encouraging, there are still some problems to be solved considering its translational application. First, the unique functions of different BETs in different stages of sepsis are still poorly understood. Second, the dosage and administration time of BETis are undefined and need to be further explored. In the late stage of sepsis, the immune system of patients may be suppressed or even paralyzed, thus necessitating an optimal, individualized therapeutic regimen. Third, since most BETis have predominant anti-cancer activity, more attention should be paid to their long-term toxicity and side effects. Fourth, developing new kinds of BETis with high efficacy and low toxicity through new technology/concept (e.g., PROTACs, lysosome-targeting chimeras [LYTACs], or phase separation) are always important for future studies.
